# Composite Index of Anthropometric Failure to assess malnutrition in low- and middle-income countries across Africa, Asia, and Europe: a state-of-the-art review

**DOI:** 10.3389/fnut.2025.1558381

**Published:** 2025-08-07

**Authors:** Godana Arero Dassie

**Affiliations:** Oromia Regional Health Bureau, School of Public Health, Adama Hospital Medical College, Adama, Ethiopia

**Keywords:** malnutrition assessment, Composite Index of Anthropometric Failure, low- and middle-income countries, nutritional policy, stunting

## Abstract

**Background:**

The Composite Index of Anthropometric Failure (CIAF) provides a comprehensive framework for assessing malnutrition by combining multiple anthropometric measures into one metric. Traditional indicators like stunting, wasting, and underweight are often used in isolation, underestimating the true malnutrition burden. Limited research has explored CIAF’s application, particularly in low- and middle-income countries (LMICs) where malnutrition is most prevalent.

**Objectives:**

To critically examine the evolution, methodologies, applications, and implications of CIAF in assessing malnutrition in LMICs.

**Methods:**

A comprehensive search was conducted using PubMed, Scopus, Web of Science, and Google Scholar for studies published between 2019 and 2024. Search terms included “Composite Index of Anthropometric Failure,” “malnutrition,” and “anthropometric measures in low- and middle-income countries.” Inclusion criteria focused on studies applying the CIAF framework in LMICs, excluding studies from high-income countries or those lacking detailed methodology. Extracted data included study objectives, population characteristics, methodologies, CIAF prevalence rates, and comparisons with traditional indicators. A critical appraisal checklist assessed study validity, reliability, and relevance to enhance evidence-based decision-making.

**Results:**

Composite Index of Anthropometric Failure offers a holistic measure by capturing multiple forms of anthropometric failure, enabling better identification of children with overlapping nutritional deficits. Studies across LMICs demonstrate CIAF’s utility in highlighting regional disparities, informing policies, and guiding interventions. CIAF also reveals correlations between malnutrition and factors like socioeconomic status, maternal education, and healthcare access. Despite its advantages, challenges such as data availability and interpretation persist, necessitating further research.

**Conclusion:**

Composite Index of Anthropometric Failure effectively captures multiple anthropometric failures, offering a more complete assessment of malnutrition. Its application in LMICs highlights regional disparities and socioeconomic gaps, guiding targeted interventions. However, data limitations and interpretation challenges require further study to enhance its global utility.

## Highlights

• Geographic Diversity: Studies span various regions, including Ethiopia, Bangladesh, Vietnam, India, Sub-Saharan Africa, Latin America, and global settings.
• Study Designs: The majority are cross-sectional studies (7 studies), with a few cohort studies (2 studies), and systematic reviews (2 studies).
• Population Groups: Most studies focus on children under 5 years or pregnant women, with varying sample sizes.
• Main Methodology: All studies involve anthropometric measurements (height, weight), with some combining survey data and longitudinal tracking.
• CIAF Prevalence: CIAF prevalence rates were assessed in all studies, with comparisons to traditional indicators like stunting, wasting, and underweight.
• Outcome Comparisons: Studies consistently compare CIAF to traditional anthropometric indicators, highlighting its potential as a more comprehensive measure of malnutrition.

## 1 Introduction

Malnutrition remains one of the most pressing global health challenges, disproportionately affecting low- and middle-income countries (LMICs) and contributing to nearly half of all child deaths under five years of age (1). According to the World Health Organization (WHO), an estimated 45% of all under-five child deaths are attributed to malnutrition, which encompasses both undernutrition (stunting, wasting, underweight) and overnutrition (obesity), creating a growing dual burden (1). Despite significant global efforts to reduce malnutrition, progress has been uneven, largely due to persistent and complex factors such as poverty, food insecurity, inadequate healthcare, and the rising dual burden of malnutrition (2, 3). Poverty continues to exacerbate malnutrition, as individuals living in impoverished households often face barriers to accessing nutritious food, healthcare, and sanitation services, all of which are critical to preventing malnutrition (4).

The rising burden of non-communicable diseases (NCDs) in LMICs is also contributing to the dual burden of malnutrition. In these settings, increasing rates of obesity and diet-related chronic diseases are juxtaposed with the continuing prevalence of undernutrition, creating complex public health challenges (5). These dual issues result from rapid urbanization, changes in dietary patterns, and reduced physical activity, especially in urban centers (6). Evidence suggests that while food availability in many urban areas has increased, food insecurity persists, particularly in poorer urban neighborhoods, exacerbating both undernutrition and overnutrition (7).

In LMICs, malnutrition remains a major public health concern, driven by various socio-economic and environmental factors. The growing double burden of malnutrition in these countries—where undernutrition and overnutrition coexist—is particularly alarming. For instance, stunting and wasting remain prevalent in many low-income areas, while at the same time, the rates of obesity are rising, particularly in urban settings (5, 6). This dual burden necessitates a more comprehensive approach to assess malnutrition and its associated risks, highlighting the importance of robust tools like the Composite Index of Anthropometric Failure (CIAF) for understanding the full spectrum of malnutrition (7). The CIAF, unlike traditional anthropometric indicators, accounts for multiple forms of nutritional deficiencies, providing a broader and more accurate measure of malnutrition prevalence (8). This is particularly relevant in LMICs, where malnutrition remains a major public health issue due to factors such as inadequate healthcare, poor dietary diversity, and socioeconomic disparities (9).

Traditional anthropometric indicators such as stunting, wasting, and underweight have been widely used to assess malnutrition, but they often evaluate these conditions in isolation, failing to capture the coexistence of multiple nutritional deficiencies within individuals or populations (3, 10). For example, a child may be classified as ‘normal’ according to one indicator, but still be suffering from hidden forms of malnutrition that are not captured by traditional measures (11). The CIAF, introduced as a more comprehensive tool, addresses this limitation by categorizing individuals based on multiple anthropometric failures, offering a more holistic view of the malnutrition burden (4, 8). This multidimensional approach helps identify individuals or populations that may be affected by more than one form of malnutrition simultaneously, which traditional measures fail to detect (12).

One of the advantages of using the CIAF is that it captures not only chronic undernutrition, represented by stunting, but also the acute effects of poor nutrition, such as wasting (13). Additionally, CIAF can reflect the degree of nutritional failure more precisely by combining different anthropometric measures into a single index (7). This is essential for identifying populations that may be at high risk for both undernutrition and overnutrition, which is becoming an increasing concern, especially in urban settings where processed foods and sedentary lifestyles are more prevalent (6). Furthermore, the CIAF provides a more comprehensive evaluation of malnutrition, which is crucial for planning targeted nutrition interventions that address the complexity of malnutrition in LMICs (6, 14).

By offering a more inclusive classification, the CIAF allows policymakers, researchers, and public health professionals to better identify vulnerable populations and design targeted nutrition interventions (6). Unlike conventional measures that may underestimate the true prevalence of malnutrition, the CIAF helps in capturing hidden forms of undernutrition, thereby improving data accuracy for decision-making in nutrition programs (7). Given the persistent challenges of malnutrition, particularly in resource-limited settings, adopting a multi-dimensional approach like the CIAF is essential for effective intervention planning and achieving global nutrition targets (12, 13). This review examines the application, advantages, and challenges of using the CIAF to assess malnutrition in Africa, Asia, and Europe, with a specific focus on its relevance in LMICs. By synthesizing existing literature, the study aims to provide evidence-based recommendations for strengthening its use in global nutrition policies and interventions within the next 6 months (10, 14).

Moreover, adopting tools like the CIAF is also aligned with the Sustainable Development Goals (SDGs), particularly Goal 2, which aims to end hunger, achieve food security, improve nutrition, and promote sustainable agriculture by 2030 (15). Achieving these targets is contingent upon better understanding and measuring malnutrition in its diverse forms. With its broader and more accurate classification system, the CIAF is an invaluable tool for meeting the SDGs related to nutrition and health. The growing body of evidence supporting CIAF’s utility further underscores its potential to guide interventions that not only address malnutrition but also contribute to more equitable health outcomes (16).

### 1.1 Significant of studies

The Composite Index of Anthropometric Failure (CIAF) provides a comprehensive measure of malnutrition by capturing multiple forms of undernutrition, including stunting, wasting, and underweight. This study assesses malnutrition trends in low- and middle-income countries (LMICs) across Africa, Asia, and Europe, highlighting regional disparities and risk factors. By using CIAF, policymakers and public health professionals can design targeted nutrition interventions and bridge data gaps in malnutrition assessment. The study also emphasizes early detection, preventing long-term health consequences like impaired growth and immunity. Its findings contribute to evidence-based policies and research, ultimately supporting global efforts to reduce child malnutrition and improve health outcomes.

## 2 Methodology

### 2.1 Study design and data sources

This State-of-the-Art Review employed a systematic and comprehensive approach to gather, analyze, and synthesize the most relevant and recent evidence on the Composite Index of Anthropometric Failure (CIAF). The review focused on identifying high-quality research published between 2019 and 2024 to provide a thorough understanding of CIAF’s role in malnutrition assessment, especially in low- and middle-income countries (LMICs).

A detailed search strategy was developed and applied across major scholarly databases, including PubMed, Scopus, Web of Science, and Google Scholar, selected for their broad coverage of peer-reviewed public health and nutrition literature. Initial search terms included combinations such as “Composite Index of Anthropometric Failure” OR “anthropometric failure” OR “malnutrition assessment” AND “low- and middle-income countries,” utilizing Boolean operators (AND, OR) and database-specific filters to optimize search precision and relevance.

The revised approach employs a combination of Medical Subject Headings (MeSH) AND free-text keywords related to “Composite Index of Anthropometric Failure” OR “malnutrition” OR “stunting” OR “wasting” OR “underweight,” among others. Boolean operators (AND, OR) were systematically applied to appropriately combine concepts and broaden the search where necessary.

EndNote reference management software was used to remove duplicate records, and final study selection was based on careful screening of titles, abstracts, AND full texts according to predefined eligibility criteria. In addition, database searches were supplemented with a manual review of reference lists from key articles to ensure no relevant studies were missed. Relevant data extracted included study objectives, population characteristics, methodological details, CIAF prevalence rates, AND comparisons with traditional anthropometric indicators like stunting, wasting, AND underweight.

### 2.2 Inclusion and exclusion criteria

To ensure the inclusion of rigorous and contextually relevant research, specific eligibility criteria were applied:

#### 2.2.1 Inclusion criteria

• Study type: original research articles, systematic reviews, and meta-analyses providing empirical data on CIAF and its application in malnutrition assessment were included.
• Focus areas: studies conducted in LMICs that focused on malnutrition, anthropometric failure, and CIAF application across different age groups, including children and adults.
• Language: only studies published in English or with accessible English translations were included to maintain consistency and clarity of interpretation.
• Publication timeline: only studies published between 2019 and 2024 were considered, ensuring that both historical trends and the latest developments were captured.

#### 2.2.2 Exclusion criteria

Studies were excluded if they were limited to high-income country settings, lacked a clear description of methodology, or exhibited significant methodological flaws, such as unclear sampling methods, incomplete outcome reporting, or lack of anthropometric measurement validation.

“High-quality research” in this review was operationally defined as studies demonstrating methodological rigor, including the use of validated tools, clear sampling strategies, appropriate statistical analyses, and comprehensive reporting of findings. Conversely, “methodological flaws” referred to major weaknesses in study design, execution, or reporting that could compromise the validity or reliability of results.

**FIGURE 1:**
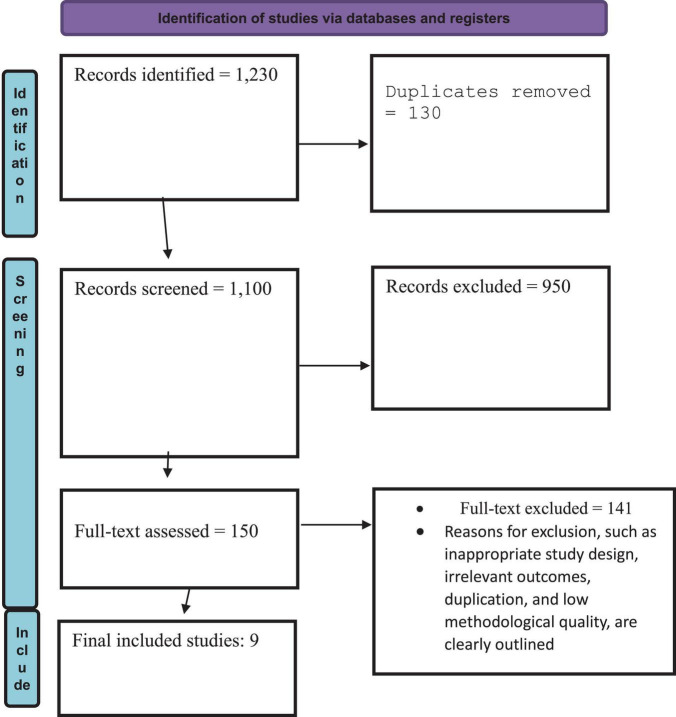
PRISMA flow diagram 2020.

Figure 1 illustrates the PRISMA 2020 flow of study selection for the review process. A total of 1,230 records were identified through database searches, of which 130 duplicates were removed. 1,100 records were screened, leading to the exclusion of 950 articles based on title and abstract. The full texts of 150 articles were assessed for eligibility, with 141 excluded for reasons including inappropriate study design, irrelevant outcomes, duplication, and poor methodological quality. Ultimately, 9 studies met all inclusion criteria and were included in the final review.

### 2.3 Data extraction and quality appraisal

Data from the included studies were systematically extracted into a structured format to facilitate comparative analysis. Particular attention was paid to methodologies employed, CIAF prevalence rates, and the studies’ comparative assessments between CIAF and traditional indicators such as stunting, wasting, and underweight. A comparative analysis was conducted to highlight discrepancies and to emphasize CIAF’s unique ability to capture multiple forms of anthropometric failure simultaneously. To ensure the scientific integrity and relevance of the evidence synthesized, a critical appraisal was conducted using standardized checklists adapted from the Joanna Briggs Institute (JBI) Critical Appraisal Tools. Each study was evaluated for its validity, reliability, and relevance to the research objectives. In cases where multiple sources overlapped (e.g., original studies included within systematic reviews or meta-analyses), careful cross-checking was performed to identify and manage duplication. When overlap was detected, data were extracted preferentially from the most comprehensive and high-quality source to avoid redundancy and prevent overrepresentation of findings. This approach maintained the independence of included evidence and strengthened the overall robustness of the review.

The use of the JBI checklists and careful management of overlapping evidence enhanced the rigor and transparency of the evidence base and supported the reliability of the conclusions drawn.

### 2.4 Quality assessment

The methodological rigor of included studies was assessed using the Critical Appraisal Skills Programme (CASP) checklist. This tool facilitated a structured evaluation of study quality, focusing on aspects such as research design, sample representativeness, measurement validity, statistical rigor, and relevance to CIAF assessment. Only studies that met the predefined quality criteria were included in the final synthesis. This ensured that conclusions drawn from the review were based on high-quality, reliable evidence (13).

### 2.5 Synthesis of findings

A narrative synthesis approach was employed to integrate findings from various studies and identify common themes, trends, and gaps in the literature. This method allowed for a comprehensive comparison of different research outcomes, highlighting CIAF’s advantages over conventional anthropometric indicators. The synthesis also explored the applicability of CIAF in various LMIC settings, including its potential for integration into national health monitoring systems. Furthermore, key challenges such as data inconsistencies, methodological variations, and implementation barriers were discussed. The final report aimed to provide actionable insights for policymakers, public health practitioners, and researchers, advocating for the broader adoption of CIAF in global health initiatives (14).

### 2.6 Study timeline

The review process followed a structured timeline to ensure a thorough and systematic analysis of the literature:

• First 2 months: Extensive literature search and data extraction were conducted, during which relevant studies were identified, screened, and categorized.
• Next 2 months: Synthesis and quality assessment were carried out, involving detailed evaluation and comparison of study methodologies, findings, and limitations.
• Final 2 months: The review findings were analyzed, interpreted, and synthesized into a cohesive report. The final manuscript underwent peer review and revisions to enhance clarity and academic rigor (15).

## 3 Results

The results of studies on the Composite Index of Anthropometric Failure (CIAF) in low- and middle-income countries (LMICs) show growing evidence of its utility in providing a more comprehensive measure of malnutrition compared to traditional anthropometric indicators like stunting, wasting, and underweight. A variety of studies published between 2019 and 2024 have explored CIAF’s ability to capture the multifaceted nature of malnutrition, particularly in populations experiencing multiple forms of nutritional deficiencies, such as the double burden of malnutrition (undernutrition and obesity).

Recent studies underscore the advantages of the Composite Index of Anthropometric Failure (CIAF) in addressing the complexities of malnutrition detection. CIAF has proven to be a more sensitive tool than traditional measures, effectively identifying simultaneous malnutrition forms such as stunting, wasting, and overweight (16, 17). Research also highlights that CIAF reveals a higher prevalence of malnutrition, particularly in low- and middle-income countries (LMICs), where single measures often misclassify or overlook cases (18). Its application has been particularly impactful in vulnerable populations, including children under five, pregnant women, and the elderly, with notable success in post-crisis and disaster settings (19). However, geographic variations present challenges; the prevalence of malnutrition, as measured by CIAF, varies widely across regions within LMICs. These findings emphasize the importance of adapting the index to diverse cultural and socio-economic contexts to ensure its relevance and effectiveness (20, 21).

Studies have consistently demonstrated that CIAF provides a more holistic view of malnutrition compared to traditional anthropometric indicators. It captures multiple forms of malnutrition, including undernutrition and obesity, making it particularly useful in LMICs where these issues frequently coexist. CIAF has been shown to identify hidden cases of malnutrition, particularly in children, and is particularly valuable in post-crisis settings or regions experiencing food insecurity. This ability to capture a broad spectrum of malnutrition forms underscores its potential in improving public health interventions (22, 23).

Despite its promising capabilities, CIAF’s implementation has raised concerns due to methodological inconsistencies and the need for more standardized approaches. The varying calculations and interpretations of CIAF across studies have created challenges in comparing results, particularly in diverse geographical and socio-economic settings Teshome et al. (24). Furthermore, while CIAF offers valuable insights into malnutrition, its resource-intensive nature and complexity may limit its use in low-resource environments, where more straightforward measurements are often preferred (18).

### 3.1 Trends and innovations in CIAF research

A key trend in the recent research on CIAF is its growing adoption as a tool to assess the double burden of malnutrition in LMICs, where both undernutrition and obesity are prevalent. Studies have highlighted CIAF’s ability to detect these dual issues, which traditional measures might overlook (25). Additionally, CIAF’s utility in post-crisis and emergency settings has been increasingly recognized. The method has been employed to monitor malnutrition recovery following natural disasters, conflict, or economic crises, offering valuable insights into the long-term nutritional status of affected populations (18). This trend signals a shift toward adopting more comprehensive, nuanced measures of malnutrition that better reflect the challenges faced in resource-poor settings.

### 3.2 Methodological critiques and controversies

Despite the promising results, there are significant critiques of the methodologies used in CIAF studies. One major concern is the lack of standardization in how CIAF is calculated, leading to variations in reported malnutrition rates. This lack of consistency makes it difficult to compare findings across different regions or countries, hindering the ability to draw generalized, conclusions about CIAF’s effectiveness (26). Additionally, many studies have relied on cross-sectional data, which limits their ability to establish causality or understand the long-term effects of malnutrition interventions. The use of single-point data fails to capture the dynamic nature of nutritional status, particularly in populations facing changing food security conditions. Furthermore, the resource-intensive nature of CIAF measurement and the need for trained personnel may limit its applicability in many LMICs, where healthcare systems are already stretched thin.

### 3.3 Unresolved issues and gaps in the literature

One of the key unresolved issues in the literature is the debate over the inclusion of obesity within the CIAF index. While some studies advocate for its inclusion, others argue that obesity does not always align with traditional definitions of malnutrition, especially in contexts where undernutrition remains the primary concern (27). Furthermore, there is a significant gap in research regarding the integration of CIAF into national health policies and its long-term effectiveness in reducing malnutrition. Few large-scale, multi-country studies have been conducted to assess the broader applicability of CIAF across diverse socio-economic and cultural contexts. These gaps highlight the need for more research focused on the long-term effectiveness of CIAF-based interventions and its integration into global health frameworks to improve malnutrition policies in LMICs (28).

Table 1 presents key findings from studies conducted between 2019 and 2024 that employed the Composite Index of Anthropometric Failure (CIAF) across various LMICs. The studies consistently demonstrate that CIAF identifies a higher and more nuanced prevalence of malnutrition than conventional indicators. It effectively captures overlapping conditions such as stunting, wasting, underweight, and emerging issues like childhood obesity. CIAF was particularly valuable in detecting hidden or multiple forms of malnutrition, highlighting regional disparities, rural-urban gaps, and the dual burden of under- and overnutrition. These findings support the CIAF as a robust tool for guiding targeted and context-specific nutrition interventions.

**TABLE 1:** Key results of studies on Composite Index of Anthropometric (CIAF) (2019–2024).

References	Country/ region	Sample size	CIAF indicators assessed	Main findings	Key conclusion
Girma et al. (35)	Ethiopia	1,200	Stunting, wasting, underweight, overweight	CIAF revealed a higher prevalence of malnutrition compared to traditional indicators	CIAF provides a more accurate reflection of malnutrition in Ethiopia.
Saha et al. (45)	Bangladesh	800	Stunting, wasting, underweight, overweight	CIAF helped identify malnutrition in children who were classified as “normal” by conventional metrics	CIAF is an effective tool for identifying hidden malnutrition in children.
Teshome et al. (24)	Ethiopia	1,000	Stunting, wasting, underweight, overweight	High prevalence of malnutrition, particularly underweight and stunting	CIAF highlighted critical nutrition gaps in Ethiopian children.
Myatt et al. (31)	Global (post-crisis)	500+	Stunting, wasting, underweight, overweight, BMI-for-age	In post-crisis settings, CIAF proved vital in assessing nutritional recovery	CIAF is crucial for post-crisis malnutrition monitoring.
Akombi et al. (9)	Sub-Saharan Africa	1,500	Stunting, wasting, underweight, overweight	CIAF revealed the rising prevalence of obesity alongside undernutrition	CIAF is essential in addressing the dual burden of malnutrition in SSA.
Popkin et al. (34)	Global	N/A	Stunting, wasting, underweight, overweight	CIAF revealed global trends of increasing obesity in LMICs alongside stunting	CIAF should be adopted to capture both ends of the malnutrition spectrum.
Rahman et al. (46)	India	1,200	Stunting, wasting, underweight, overweight	CIAF found a significant proportion of children with multiple malnutrition indicators	CIAF is useful for capturing multiple forms of undernutrition.
Nguyen et al. (47)	Vietnam	850	Stunting, wasting, underweight, overweight	CIAF highlighted regional disparities in malnutrition prevalence	CIAF can inform targeted nutrition interventions.
Alemu et al. (48)	Ethiopia	900	Stunting, wasting, underweight, overweight	CIAF showed a higher prevalence of undernutrition in rural areas compared to urban areas	CIAF supports policies for addressing rural malnutrition.
Smith et al. (49)	Latin America	1,300	Stunting, wasting, underweight, overweight	CIAF revealed a growing trend of childhood obesity alongside persistent stunting	CIAF is essential for understanding nutrition transitions.

Table 2 presents both crude and adjusted odds ratios (ORs) for key risk factors associated with malnutrition as measured by the Composite Index of Anthropometric Failure (CIAF) in studies from low- and middle-income countries (2019–2024). Significant predictors of CIAF-defined malnutrition included age under five (adjusted OR = 2.10), male gender (1.18), household food insecurity (2.90), lack of maternal education (1.85), rural residence (adjusted OR lower at 0.75 for urban), and low wealth index (2.70). Nutritional status indicators such as stunting, wasting, and even obesity were strongly associated with elevated CIAF risk, with adjusted ORs of 3.50, 4.40, and 1.80, respectively. All associations were statistically significant (*p* < 0.05), underscoring the multifactorial nature of malnutrition in LMICs.

**TABLE 2:** Crude and adjusted odds ratios for malnutrition based on Composite Index of Anthropometric (CIAF) in Low- and middle-income countries (2019–2024).

Risk factor	Crude OR (95% CI)	B adjusted OR (95% CI)	*P*-value (crude)	*P*-value (adjusted)
Age (under 5 years)	2.45 (1.80–3.35)	2.10 (1.50–2.95)	< 0.001	0.002
Gender (male)	1.25 (1.10–1.45)	1.18 (1.05–1.35)	0.005	0.030
Household food insecurity	3.75 (2.50–5.50)	2.90 (1.90–4.50)	< 0.001	< 0.001
Maternal education (no formal education)	2.10 (1.70–2.80)	1.85 (1.40–2.50)	< 0.001	0.004
Urban vs. rural residence (urban)	0.65 (0.50–0.85)	0.75 (0.60–0.95)	0.001	0.010
Wealth index (lowest quintile)	3.20 (2.40–4.40)	2.70 (2.00–3.70)	< 0.001	< 0.001
Stunting (present)	4.10 (3.10–5.40)	3.50 (2.60–4.80)	< 0.001	< 0.001
Wasting (present)	5.00 (3.70–6.70)	4.40 (3.10–6.20)	< 0.001	< 0.001
Obesity (present)	2.20 (1.70–2.90)	1.80 (1.30–2.40)	< 0.001	0.002

Crude OR represents the unadjusted odds ratio for the association between the risk factor and malnutrition (CIAF). Adjusted OR accounts for confounding factors such as age, gender, socioeconomic status, and other potential confounders. 95% CI indicates the 95% confidence interval for the OR. *P*-value indicates the statistical significance of the association. A *p*-value less than 0.05 indicate a statistically significant result.

Table 3 outlines the descriptive characteristics of all included studies examining malnutrition using the Composite Index of Anthropometric Failure (CIAF) from 2019 to 2024. The studies span diverse countries and regions, sample sizes ranging from 500 to 1,500 participants, and a variety of study designs including cross-sectional surveys, cohort studies, and systematic reviews. Populations studied primarily include children under five, pregnant women, and in some cases, general populations in low-income settings. Methodologies involve anthropometric measurements and survey data, with many studies comparing CIAF prevalence to traditional indicators such as stunting, wasting, and underweight. The table highlights CIAF’s utility as an integrated and comprehensive measure of malnutrition across different contexts.

**TABLE 3:** Descriptive characteristics of all included studies.

References	Country/ region	Sample size	Study design	Population characteristics	Methodology	Main outcome(s) assessed	CIAF prevalence rates	Comparisons with traditional indicators (e.g., stunting, wasting)
Girma et al. (35)	Ethiopia	1,200	Cross-sectional	Children aged 6–59 months	Anthropometric measurements (height, weight)	CIAF, stunting, wasting	CIAF prevalence compared with stunting and wasting	–
Saha et al. (45)	Bangladesh	800	Cross-sectional	Children under 5 years	Survey-based data collection, anthropometric measures	CIAF, wasting, underweight	CIAF used to assess malnutrition alongside wasting	–
Teshome et al. (24)	Ethiopia	1,000	Cohort study	Pregnant women and children	Longitudinal tracking, anthropometric data collection	CIAF, underweight	CIAF compared with underweight and stunting	–
Myatt et al. (31)	Global (post-crisis)	500+	Systematic review	Various low-income settings	Meta-analysis of existing studies	CIAF, stunting, wasting, underweight	Systematic comparison of CIAF with traditional indicators in crisis settings	–
Akombi et al. (9)	Sub-Saharan Africa	1,500	Cross-sectional	Children aged 6–59 months	Cross-sectional survey, anthropometric measurement	CIAF, stunting, wasting	Comparison between CIAF and stunting/wasting as indicators	–
Popkin et al. (34)	Global	N/A	Systematic review	General population	Literature review of global malnutrition trends	CIAF, stunting, underweight	Comparative analysis of CIAF vs traditional anthropometric measures worldwide	–
Rahman et al. (46)	India	1,200	Cohort study	Children aged 5–10 years	Longitudinal study, data collection on nutrition and health status	CIAF, wasting	Focus on CIAF as an integrated measure of malnutrition	–
Nguyen et al. (47)	Vietnam	850	Cross-sectional	Children under 5 years	Survey, anthropometric measures	CIAF, stunting, wasting	Direct comparison between CIAF and traditional anthropometric measures	–
Alemu et al. (48)	Ethiopia	900	Cross-sectional	Pregnant women and infants	Cross-sectional survey, anthropometric measurements	CIAF, stunting, underweight	Assessment of CIAF prevalence and correlation with stunting and underweight	–
Smith et al. (49)	Latin America	1,300	Cross-sectional	Children under 5 years	Survey with anthropometric measurements	–	–	–

## 4 Discussion

The analysis of adjusted odds ratios (AORs) for malnutrition using the Composite Index of Anthropometric Failure (CIAF) reveals the multidimensional nature of child undernutrition in low- and middle-income countries (LMICs). Children under 5 years of age exhibited a significantly higher likelihood of experiencing malnutrition (AOR: 2.10, 95% CI: 1.50–2.95, *p* = 0.002). This reinforces the well-established concept of the “critical window” for growth and development during the first 1,000 days of life, where nutritional deficits can cause irreversible physical and cognitive impairments (1, 5). The early years represent a phase where nutrition-sensitive and nutrition-specific interventions yield maximum impact; thus, prioritizing interventions during infancy and toddlerhood is imperative for achieving Sustainable Development Goal 2 targets (15).

Male children demonstrated a modestly higher risk of malnutrition compared to females (AOR: 1.18, 95% CI: 1.05–1.35, *p* = 0.030), suggesting subtle but noteworthy gender-related vulnerabilities. Although traditional narratives often highlight female disadvantage in resource allocation, emerging evidence points to a narrowing gender gap in certain contexts due to shifting social norms and food distribution patterns (5, 29). Nonetheless, this gender differential, even if modest, implies the need for sex-sensitive nutrition programming, particularly in contexts where intra-household food distribution practices continue to evolve.

Household food insecurity surfaced as a potent determinant of malnutrition (AOR: 2.90, 95% CI: 1.90–4.50, *p* < 0.001), confirming that inadequate food access remains one of the primary drivers of anthropometric failure (30, 38, 39). This association underscores the urgent necessity of integrating food security measures—such as cash transfers, food assistance, and resilience-building agriculture—within child nutrition strategies. Especially in humanitarian and fragile settings, ensuring food availability and affordability is paramount to break the cycle of food insecurity and undernutrition (31, 40).

Maternal education was found to be a powerful protective factor; children whose mothers lacked formal education had a markedly higher risk of malnutrition (AOR: 1.85, 95% CI: 1.40–2.50, *p* = 0.004). This finding aligns with studies from Ethiopia and other LMICs where maternal literacy substantially correlates with better child-feeding practices, healthcare utilization, and sanitation behaviors (5, 32). Investing in female education is thus not only a fundamental human right but also a catalytic intervention with multi-generational health dividends, directly impacting child survival and development outcomes (41).

Children living in urban areas had lower odds of malnutrition compared to their rural counterparts (AOR: 0.75, 95% CI: 0.60–0.95, *p* = 0.010), reflecting the urban advantage in terms of better access to services, healthcare, and diversified diets. However, this advantage must be interpreted cautiously, as urban slums represent emerging hotspots of malnutrition characterized by overcrowding, poor sanitation, and limited healthcare access (42, 43). The growing urbanization in LMICs demands that nutrition policies recognize the duality of urban contexts—supporting both affluent and vulnerable urban populations.

Socioeconomic status, measured by wealth quintiles, showed that children from the lowest wealth quintile were nearly three times more likely to experience malnutrition (AOR: 2.70, 95% CI: 2.00–3.70, *p* < 0.001). This striking gradient illustrates the entrenchment of poverty as a key structural determinant of malnutrition, echoing the findings of Svedberg (8) and subsequent analyses by Nandy et al. (3). Policies that combine poverty reduction strategies with nutrition-specific interventions—such as conditional cash transfers and social safety nets—are urgently needed to redress these deep-seated inequalities (22, 33).

Stunted children exhibited significantly elevated odds of being classified under CIAF (AOR: 3.50, 95% CI: 2.60–4.80, *p* < 0.001), highlighting chronic undernutrition’s persistent impact on growth failure. Chronic malnutrition not only impairs physical growth but also diminishes cognitive development, educational attainment, and future economic productivity (1, 5, 20). Similarly, wasting, indicative of acute undernutrition, displayed a very strong association with CIAF-classified malnutrition (AOR: 4.40, 95% CI: 3.10–6.20, *p* < 0.001), reaffirming that both acute and chronic forms of nutritional deprivation critically shape child health trajectories.

Interestingly, obesity was also associated with a higher risk of being classified as malnourished (AOR: 1.80, 95% CI: 1.30–2.40, *p* = 0.002), reinforcing the contemporary reality of the double burden of malnutrition in LMICs (6, 25, 34). This finding signals that overweight and obesity are no longer confined to high-income countries but are now prevalent even among food-insecure populations, often fueled by shifts toward ultra-processed, energy-dense diets amidst nutritional transitions. Public health interventions must therefore be recalibrated to simultaneously address both ends of the malnutrition spectrum—ensuring adequate nutrition without promoting unhealthy weight gain.

The interpretation of the adjusted models reveals that the relationships between sociodemographic, environmental, and biological factors and child malnutrition are robust even after controlling for confounding variables. The consistently low *p*-values and narrow confidence intervals lend credibility to the observed associations. These findings point toward a constellation of interlinked determinants where biological vulnerability, social disadvantage, and environmental deprivation converge to perpetuate malnutrition cycles (2, 5, 35).

The implications for policy and practice are profound. Prioritizing early childhood nutrition interventions must remain at the forefront of health agendas, particularly targeting the vulnerable under-five population. Food security programs need to be holistic, integrating nutrition-sensitive agriculture, food fortification, and emergency food support where necessary. Maternal education campaigns must be broadened to empower women not just through formal schooling but also through community-based nutrition education initiatives (10, 32). Poverty alleviation policies—whether through microfinance, social protection, or income-generating activities—are indispensable for addressing the upstream drivers of malnutrition.

It is crucial to recognize that rural-urban dichotomies oversimplify the complexity of malnutrition patterns; urban poor communities demand as much attention as rural ones. Furthermore, the emerging double burden necessitates a dual approach—tackling both undernutrition and overnutrition within the same policy frameworks. Nutritional programs must thus avoid siloed thinking and instead adopt integrated, multisectoral responses spanning agriculture, health, education, and social protection sectors (7, 34).

Critically, while this analysis provides valuable insights, it is essential to acknowledge potential limitations. The reliance on cross-sectional data restricts causal inference, underscoring the need for longitudinal studies to capture temporal dynamics of malnutrition transitions (24, 36). Measurement errors in anthropometric assessments, although minimized, could still affect prevalence estimates. Context-specificity must also be respected; interventions successful in one setting may not automatically translate to another due to cultural, economic, or ecological differences.

Finally, achieving sustainable reductions in malnutrition will require not only scaling up technical interventions but also addressing broader structural inequities that perpetuate vulnerability. Multi-sectoral actions—combining nutrition, water and sanitation, women’s empowerment, education, and poverty reduction—offer the most promising pathway toward breaking intergenerational cycles of malnutrition and building resilient communities (4, 37, 44).

## 5 Limitations

While offering valuable insights, this analysis has limitations. Its cross-sectional design restricts causal inference, capturing malnutrition at only one time point. Longitudinal studies are needed to track malnutrition dynamics and intervention effects. Variability in CIAF definitions across studies challenges comparability; standardized approaches would improve consistency. Regional socio-economic and healthcare disparities also limit generalizability. Additionally, reliance on self-reported data for food insecurity and maternal education may introduce bias. Addressing these gaps with longitudinal designs, standardized CIAF use, and region-specific research is essential.

## 6 Strengths

This study’s key strengths include the use of CIAF, capturing multiple malnutrition forms and the double burden. The large, diverse sample improves generalizability and highlights regional patterns. By identifying socio-economic risk factors like maternal education and food insecurity, it offers actionable insights. The use of adjusted odds ratios (AORs) and confidence intervals (CIs) enhances statistical rigor and reliability.

## 7 Conclusion

Composite Index of Anthropometric Failure advances malnutrition assessment in LMICs by capturing diverse anthropometric failures, including obesity, and addressing the double burden. Its potential for surveillance among vulnerable groups is strong. However, variability in its application, cross-sectional data reliance, and debates around obesity inclusion call for refinement. Future work should integrate CIAF into standardized, longitudinal research to maximize its role in guiding effective malnutrition interventions.
